# Th17 and Th17/Treg ratio at early HIV infection associate with protective HIV-specific CD8^+^ T-cell responses and disease progression

**DOI:** 10.1038/srep11511

**Published:** 2015-06-23

**Authors:** Juliana Falivene, Yanina Ghiglione, Natalia Laufer, María Eugenia Socías, María Pía Holgado, María Julia Ruiz, Cynthia Maeto, María Inés Figueroa, Luis D. Giavedoni, Pedro Cahn, Horacio Salomón, Omar Sued, Gabriela Turk, María Magdalena Gherardi

**Affiliations:** 1 Instituto de Investigaciones Biomédicas en Retrovirus y SIDA (INBIRS), Universidad de Buenos Aires-CONICET, Buenos Aires, Argentina; 2 Fundación Huésped, Buenos Aires, Argentina; 3 Hospital J.A. Fernández, Buenos Aires, Argentina; 4 Department of Virology and Immunology, Southwest National Primate Research Center, Texas Biomedical Research Institute, San Antonio, TX, USA

## Abstract

The aim of this study was to analyze Th17 and Treg subsets and their correlation with anti-HIV T-cell responses and clinical parameters during (acute/early) primary HIV infection (PHI) and up to one year post-infection (p.i). Samples from 14 healthy donors (HDs), 40 PHI patients, 17 Chronics, and 13 Elite controllers (ECs) were studied. The percentages of Th17 and Treg subsets were severely altered in Chronics, whereas all HIV-infected individuals (including ECs) showed Th17/Treg imbalance compared to HDs, in concordance with higher frequencies of activated CD8^+^ T-cells (HLA-DR^+^/CD38^+^). Better clinical status (higher CD4 counts, lower viral loads and activation) was associated with higher Th17 and lower Treg levels. We found positive correlations between Th17 at baseline and anti-HIV CD8^+^ T-cell functionality: viral inhibitory activity (VIA) and key polyfunctions (IFN-γ^+^/CD107_A/B_^+^) at both early and later times p.i, highlighting the prognostic value of Th17 cells to preserve an effective HIV T-cell immunity. Th17/Treg ratio and the IL-17 relative mean fluorescence intensity (rMFI of IL-17) were also positively correlated with VIA. Taken together, our results suggested a potential link between Th17 and Th17/Treg ratio with key HIV-specific CD8^+^ T-cell responses against the infection.

Despite medical and scientific efforts made over the past 30 years, HIV infection continues to be a major global public health concern. The mechanisms and immune system components that contribute to the natural control of the infection and disease progression in some HIV-infected persons, in contrast to the vast majority of patients that undergo rapid progression, are not fully elucidated. The discovery of these key components and their interactions during HIV infection remains a major goal in the field that could allow the design of new approaches to control and perhaps even eradicate the disease.

Th17 cells are a CD4^+^ T-cell subset, of a lineage different from Th1 and Th2^1^,^2^. They are characterized by interleukin 17 (IL-17) production and play key roles in protective inflammatory mucosal responses against bacteria and fungi, as well as in mucosal barrier integrity and homeostasis^3^,^4^,^5^,^6^. Recent studies have demonstrated that SIV and HIV infections lead to a selective depletion of Th17 cells in both blood and gastrointestinal lymphoid tissues that can predict disease progression^7^,^8^. Even more, several publications highlighted the importance of the Th17/Treg ratio in the progressive disease developed during HIV-1 and SIV infections^9^. During chronic infection it has been shown that the loss of Th17/Treg balance associates with disease progression in individuals with typical progression in contrast to ECs^10^.

All these previous studies indicate that both Th17 cells and the Th17/Treg ratio have a critical role during HIV-1 infection. However, an evaluation of the possible correlations between these parameters and the HIV-specific antiviral adaptive T-cell response is still needed.

In a previous study our group demonstrated that, during PHI, the early relative immunodominance of Gag-specific CD8^+^ T-cells was associated with CD4^+^ T-cell count preservation, in consonance with Gag immunodominance in ECs and “viremic controllers”^11^, linking the antiviral CD8^+^ T-cell response with the natural control of disease progression.

In this context, and in light of the evidence pointing to the relevance of Th17 and Treg subsets during HIV infection and AIDS progression, we hypothesized that preservation of the Th17 sub-population and Th17/Treg ratio are determinant immune factors that could impact the HIV-specific CD8^+^ antiviral response, and hence disease progression. Therefore, the aim of the present study was to perform an in depth evaluation of the dynamics of Th17 cells and Th17/Treg ratio at different stages of HIV infection, and to investigate the correlations between these parameters and markers of disease progression and the antiviral CD8^+^ T-cell functions previously associated with protection.

For the first time we demonstrated that, during PHI, higher Th17 levels directly correlate with more potent HIV antiviral T-cell responses associated with protection. Remarkably, we verified that baseline proportions of Th17 cells may have a possible prognostic value for the functional anti-HIV T-cell responses detected at later times p.i.

## Results

### Clinical characteristics of the HIV-infected individuals enrolled

The different groups of HIV-infected participants selected to perform the present study were: a group of 40 individuals diagnosed during PHI (HIV seroconversion and/or within 6 months from presumed date of infection, 95% of them corresponded to Fiebig stages V and VI^12^), 17 typical chronically infected patients (Chronics), and a group of 13 infected individuals defined as ECs. These two last groups were included as control groups in order to compare the different parameters to be evaluated in relation to those found in the PHI cohort. All the patients enrolled were ART naïve at the time of sample collection (detailed inclusion criteria for each group are defined in Materials and Methods).

A description of the clinical characteristics of the different HIV-infected groups is summarized in Table 1. For PHIs, the median estimated time p.i was 75 days (day at which baseline sample was obtained), whereas 330 days was the median day p.i. of the “one year” follow-up sample. For some analyses, PHIs were further divided into two sub-groups taking into account whether their CD4 counts dropped below 350 cells/μl, or not, at any time during the first year p.i, denoted as rapid (RPs) and typical (TPs) progressors, respectively. Clinical differences between TPs and RPs were observed at baseline. Thus, significant higher CD4 counts (p < 0.0001) and CD4/CD8 ratios (p < 0.0001) were found in TPs. Also, mean Log_10_ viral load (VL) from RPs tended to be higher than that seen in TPs (p = 0.0713). These differences showed the same trend at one year p.i, although caution must be taken due the limitation imposed by the limited number of RP samples at this time point.

Total PHIs (at both baseline and one year p.i), showed significant higher CD4 counts and CD4/CD8 ratios in comparison with Chronics (*p* values < 0.001). As expected, ECs had preserved CD4 counts compared to Chronics (p < 0.001) and higher CD4/CD8 ratios compared to both Chronics (p < 0.001) and PHIs (baseline and on year p.i; *p* values < 0.03).

### Th17/Treg ratio was diminished since early times post-infection whereas Treg and Th17 frequencies were severely altered at advanced stages of HIV progressive infection

The first aim of the study was to analyze the Th17 and Th17/Treg changes that occur during PHI infection, contrasting the variation of these parameters in PHI patients with those observed in HDs and in chronically infected persons defined as typical Chronics and ECs.

**Figure 1:**
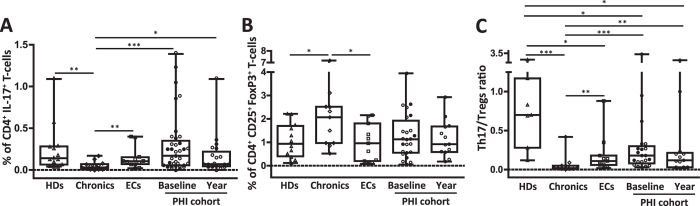
Alteration of Th17 and Treg subsets and Th17/Treg ratio at different stages of HIV infection. PBMCs were stimulated for 6 hours with anti-CD3/anti-CD28 or medium alone (background control) prior to intracellular staining. Background subtracted values of CD3^+^ CD4^+^ IL-17^+^ producing cells (Th17) are shown (**A**). Intranuclear staining for detection of CD3^+^ CD4^+^ CD25^+^ FoxP3^+^ cells (Tregs, **B**). Th17/Treg ratio was calculated for each patient (**C**). Boxes indicate median values with 25–75 percentiles and bars show the maximum and minimum values. Symbols represent individual patients: Healthy donors (Δ, HDs), Chronics (◊), Elite controllers (□, ECs) and primary HIV infection (PHI) cohort at baseline and one year p.i follow up (◦, typical progressors or TPs with CD4 counts above 350 cells/μl during the first year p.i; ∑, rapid progressors or RPs with CD4 counts below 350 cells/μl during the first year p.i). The *p* values obtained are depicted as * p < 0.05, ** p < 0.01 and *** p < 0.001.

In Fig. 1A it can be seen that the median percentage (IQR) of Th17 cells present in PHIs (both at baseline and one year follow-up, medians of 75 and 330 days p.i, respectively) were significantly higher compared to Chronics [0.17% (0.06–0.35) at baseline and 0.07% (0.05–0.22) at one year p.i in PHIs vs. 0.03% (0.01–0.07) in Chronics; *p* values 0.0002 and 0.0145, respectively]. Within the PHI cohort, a trend to a decrease in Th17 levels was observed with the advancement of the infection [0.17% (0.06–0.35) at baseline vs. 0.07% (0.05–0.22) at one year; p = 0.11]; however, differences did not reach significance probably due to the high data dispersion. Interestingly, no significant differences were found between PHIs at baseline and both HDs [0.14% (0.07–0.28)] and ECs [0.11% (0.07–0.15)]. As expected, Chronics showed the lowest Th17 levels. When analysis was done taking into account the absolute Th17 counts (number of cells/μl) similar and enhanced differences between groups were observed (data not shown).

Conversely, the percentage of Treg cells found in Chronics was higher compared to ECs and HDs [2.07% (0.97–2.52) in Chronics vs. 0.96% (0.20–1.81) in ECs and 0.94% (0.41–1.70) in HDs; *p* values 0.0356 and 0.0392, respectively; Fig. 1B]. Despite PHIs showed Treg frequencies similar to those observed in HDs and ECs [1.13% (0.56–1.93) in PHIs at baseline and 0.92% (0.59–1.68) at one year p.i], no significant differences were observed compared to Chronics. When the Th17/Treg ratio was analyzed (Fig. 1C), all HIV-infected groups showed significant differences compared to HDs [0.70 (0.28–1.17); all *p* values < 0.02], including ECs. It is worth noting that data comparing Th17/Treg ratio of ECs versus (vs.) HDs is scaring in the literature.To highlight, the ratio between these two CD4^+^ T-cell subsets was severely reduced in Chronics [0.02 (0.01–0.05)], compared to ECs [0.11 (0.06–0.18); p = 0.0054] and the PHI cohort at both baseline [0.18 (0.08–0.30); p = 0.0004] and one year p.i follow-up [0.12 (0.03–0.22); p = 0.0058].

The results described above indicated that HIV infection has a negative impact in the Th17/Treg ratio, despite natural HIV control (ECs). We also corroborated for the patient cohort of the present study that percentage of Th17 and Treg cells were severely altered in chronically infected patients with a typical progression pattern.

### Baseline Th17 cell levels were associated with immune T-cell activation and rates of disease progression

Immune activation constitutes a hallmark of HIV infection, and has been reported to occur at early times p.i^13^,^14^, thus our next aim was to analyze its relation with Th17 and Treg subsets at early stages (75 days p.i) of HIV infection, comparing individuals with different rates of immune deterioration (denoted as RPs and TPs).

**Figure 2:**
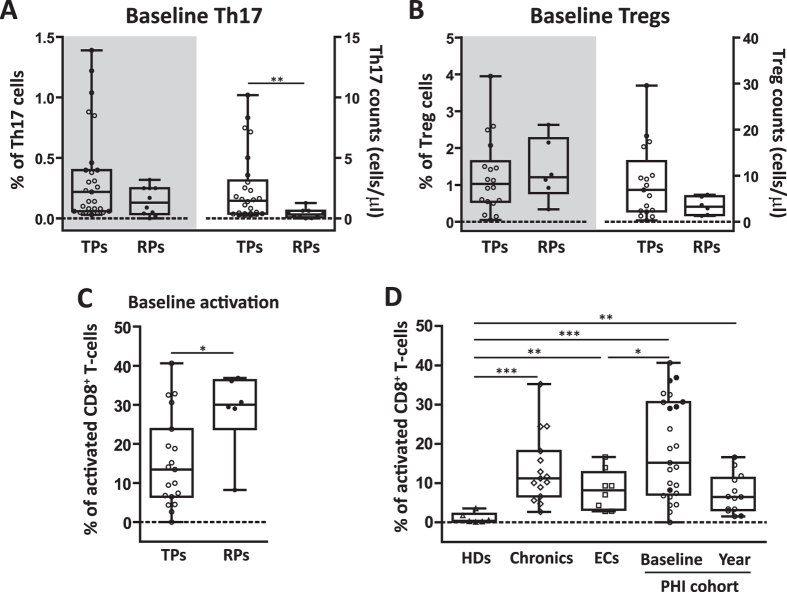
Th17 and Treg subsets among recently HIV-infected individuals that show different patterns of immune activation and disease progression. Comparison of baseline frequencies (right *y* axes) and absolute counts (left *y* axes) of Th17 (A) and Treg (B) cells between typical (TP, white symbols) and rapid (RP, black symbols) progressors. Baseline frequency of CD3^+^ CD8^+^ T-cells positive for CD38^+^ and HLA-DR^+^ activation markers was determined in TP and RP PHI sub-groups by flow cytometry (**C**). Frequency of activated CD8^+^ T-cells was determined for all groups (**D**). Boxes indicate median values with 25–75 percentiles and bars show the maximum and minimum values. Symbols represent individual patients within each group. The *p* values obtained are depicted as * p < 0.05, ** p < 0.01 and *** p < 0.001.

First, we compared both baseline proportions and absolute counts of Th17 cells that were present in PHIs with a rapid immune deterioration (RPs) compared to those that showed a more typical progression pattern (TPs). Thereby, a trend to lower percentages of Th17 cells was found in RPs [0.13% (0.04–0.25) vs. 0.22% (0.06–0.40) in TPs; p = 0.13; Fig. 2A left *y* axis], in which also a significant lower baseline median absolute number of Th17 cells was evident [0.33 (0.10–0.63) cells/μl vs. 1.47 (0.40–3.14) cells/μl in TPs; p = 0.0040; Fig. 2A right *y* axis]. When the same analysis was extended to the Treg compartment, no significant differences were found [i.e Treg counts: 6.92 (2.26–13.17) cells/μl in TPs vs. 3.27 (1.43–5.53) cells/μl in RPs; p = 0.24; Fig. 2B]. Importantly, when the levels of CD8^+^ T-cell activation were analyzed in these two sub-groups, higher frequencies of activated CD8^+^ T-cells were found in RPs [30.1% (23.8–36.3) vs. 13.5% (6.5–23.8) in TPs; p = 0.0450; Fig. 2C]. We also corroborated that, as expected, HIV infection resulted in higher levels of CD8^+^ T-cell activation since early times p.i [15.2% (7.1–30.6) in baseline PHI samples] compared to both ECs [8.2% (3.2–12.8); p = 0.0460] and normal values found in HDs [0.38% (0.18–2.19); p = 0.0007; Fig. 2D]. And at one year p.i, although not significant, immune activation tended to decrease [6.5% (3.2–11.3) in one year p.i PHI samples]. Data from this part of the study indicated that, not only Chronics but also ECs showed higher immune activation levels than HDs (Fig. 2D), in concordance with their low Th17/Treg ratio (Fig. 1C).

### Preservation of the Th17 subset positively correlated with improved clinical parameters of disease progression whereas Treg cells associated with higher viral loads and CD8^+^ T-cell activation

Our next aim was to analyze the relationships between these T-cell sub-populations and variables of disease progression in the different groups of patients. In order to perform a more exhaustive evaluation of the Th17 subset, not only frequencies and absolute counts, but also IL-17 relative mean fluorescence intensity (rMFI) and plasma IL-17 levels (as parameters of Th17 functionality) were determined.

**Figure 3:**
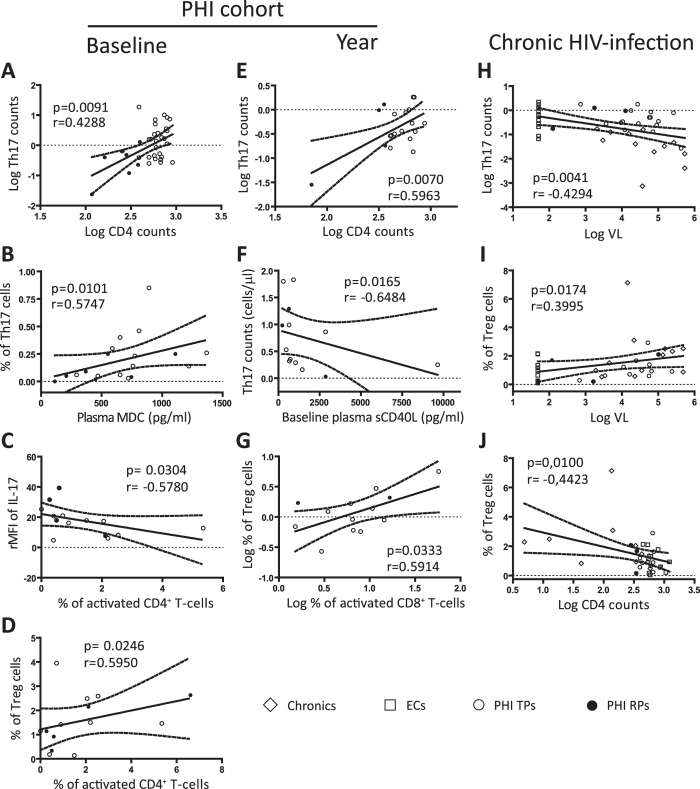
Correlations between Th17 and Treg subsets with clinical parameters of disease progression. Correlations found among PHIs at baseline: Log CD4 counts versus (vs.) Log Th17 counts (**A**); plasma macrophage-derived chemokine (MDC) levels vs. % of Th17 cells (**B**); % of activated CD4 T-cells vs. relative mean fluorescence intensity (rMFI) of IL-17 (**C**) and % of Treg cells (**D**). Correlations found among PHIs at one year p.i follow up: Log CD4 counts vs. Log Th17 counts (**E**); baseline plasma soluble CD40 ligand (sCD40L) levels vs. Th17 counts at one year p.i (**F**); Log % of activated CD8 T-cells vs. Log % of Treg cells (**G**). Relationships present at chronic stages of HIV infection (Chronic, EC and PHI one year p.i samples): Log VL vs. Log Th17 counts (**H**) and % of Treg cells (**I**); Log CD4 counts vs. % of Treg cells (**J**). Soluble plasma proteins were determined by Luminex, for details see Materials and Methods. Symbols distinguish individual patients from the different groups indicated in the figure. *ECs*: Elite controllers. *PHI*: primary HIV infection cohort. *TPs*: typical progressors. *RPs*: rapid progressors. All *r* and *p* values shown correspond to Spearman’s correlations.

When we analyzed PHIs at baseline, a positive correlation between Th17 levels and CD4 counts (p = 0.0091 r = 0.4288; Fig. 3A) was observed, in concordance with a direct correlation between IL-17 plasma levels and CD4 counts (p = 0.0308 r = 0.4960; data not shown). Interestingly, a significant positive correlation was found between percentage of Th17 cells and plasma levels of the macrophage-derived chemokine (MDC; p = 0.0101 r = 0.5747; Fig. 3B), reinforced by another positive association of this chemokine with the rMFI of IL-17 (p = 0.0360 r = 0.4960; data not shown). Of note, MDC has been previously characterized to have HIV-suppressive activities^15^. On the other hand, the percentage of activated CD4^+^ T-cells was found to be inversely correlated with the rMFI of IL-17 (p = 0.0304 r = −0.5780; Fig. 3C) and, in contrast, directly associated with the percentage of Treg cells (p = 0.0246 r = 0.595; Fig. 3D).

A similar scenario emerged at one year p.i for the PHI cohort, evidenced by the association of high Th17 levels and better immune status of the patients (CD4 counts; p = 0.0070 r = 0.5963; Fig. 3E), and the direct relation observed between Treg cells and CD8^+^ T-cell activation (p = 0.0333 r = 0.5914; Fig. 3G). Notably, levels of Th17 present at later times p.i were inversely correlated with baseline plasma quantities of the inflammatory soluble mediator CD40 ligand (sCD40L; p = 0.0165 r = −0.6484; Fig. 3F), a biomarker associated with disease progression during HIV/AIDS^16^.

When all chronically HIV-infected subjects were included in the analysis (Chronics, ECs and one year p.i samples from the PHI cohort), VL was found to be negatively correlated with Th17 counts (p = 0.0041 r = −0.4294; Fig. 3H) and directly associated to Treg levels (p = 0.0174 r = 0.3995; Fig. 3I). This last observation was in concordance with the associations of higher Treg frequencies with lower CD4 counts (p = 0.0100 r = −0.4423; Fig. 3J) and, also, with higher activated CD8^+^ T-cell counts (p = 0.0194 r = 0.4176; data not shown).

Overall these results suggest that the preservation of the Th17 subset is positively linked with an improved clinical status of the patients, in terms of CD4 counts, VL, soluble markers of disease progression and immune activation. On the contrary, the Treg subset expansion is directly associated with increased immune activation, viral replication and lower CD4 counts.

### Higher Th17 baseline levels were related to improved HIV-specific CD8^+^ T-cell functionality

**Figure 4:**
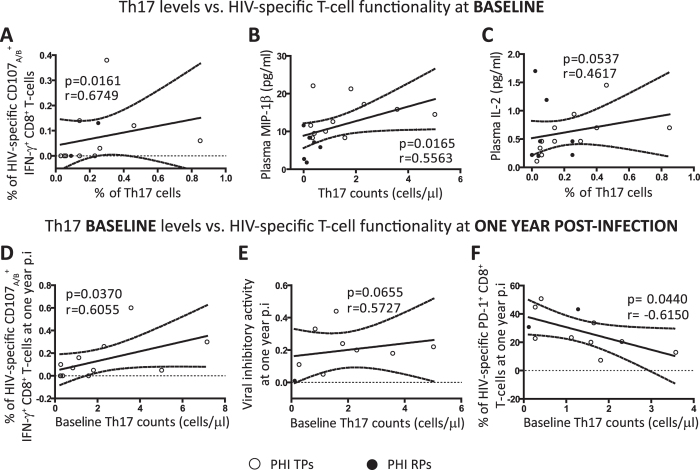
Correlations between Th17 baseline levels and HIV-specific CD8 T-cell responses previously associated with protection. Positive correlations found within PHI cohort at baseline: % of Th17 cells versus (vs.) % of HIV-specific CD107_A/B_^+^ IFN-γ^+^ CD8 T-cells (**A**) and plasma IL-2 levels (**C**); Th17 counts vs. plasma MIP-1β levels (**B**). Correlations between baseline Th17 levels and anti-HIV specific responses at later times p.i within PHI group: baseline Th17 counts vs. % of HIV-specific CD107_A/B_^+^ IFN-γ^+^ CD8 T-cells (**D**), viral inhibitory activity (**E**) and % of HIV-specific PD-1^+^ CD8 T-cells all at one year p.i (**F**). Soluble plasma proteins were determined by Luminex. HIV-specific CD8 functionality was determined with different assays: one of them allowed the evaluation of CD8 T-cells with the capacity to degranulate and simultaneously secrete IFN-γ upon HIV-peptides stimulation by flow cytometry (**A** and **D**), other measured the overall CD8 T-cell capacity to inhibit *in vitro* HIV-1 replication in autologous CD4 T-cells (**E**), and the last one measured the expression of the exhaustion marker PD-1 on HIV-specific CD8 T-cells determined upon HIV-peptides stimulation by flow cytometry (**F**). These assays are described in detail in our previous publications^11^,^48^. Symbols distinguish individual patients from the different sub-groups indicated in the figure. *PHI*: primary HIV infection cohort. *TPs*: typical progressors. *RPs*: rapid progressors. All *r* and *p* values correspond to Spearman’s correlations.

One of the major objectives of the present study was to analyze the potential impact that the proportions of Th17 and Tregs and alterations in their balance could have on the specific CD8^+^ T-cell responses with documented relevance on the antiviral HIV immunity. Therefore, our next aim was to analyze if baseline Th17 levels were related with specifc CD8^+^ T-cell functions previously associated with better clinical prognosis during HIV infection^11^. A significant positive correlation was observed between Th17 frequencies and proportions of HIV-specific CD107_A/B_^+^ IFN-γ^+^ CD8^+^ T-cells (p = 0.0161 r = 0.6749; Fig. 4A). Interestingly, in concordance with the hypothesis that a better T-cell immunity would be present in those patients with higher proportion of baseline Th17 cells,we found associations between the preservation of this subset with higher MIP-1β (T-cell chemokine associated with anti-viral properties; p = 0.0165 r = 0.5563; Fig. 4B) and also a trend to higher IL-2 plasma levels (T-cell homeostasis cytokine; p = 0.0537 r = 0.4617; Fig. 4C).

Remarkably, baseline proportions of Th17 cells suggested a possible prognostic value for the functional antiviral T-cell responses detected at later times p.i (year samples). Thus, baseline Th17 counts were positively correlated with the proportion of HIV-specific bifunctional CD8^+^ T-cells (positive for IFN-γ and CD107_A/B_) at later times (p = 0.0370 r = 0.6055; Fig. 4D). Although not significant, a trend was also found between baseline Th17 counts and the viral inhibitory activity (VIA) against *in vitro* HIV growth exerted by CD8^+^ T-cells measured in one year samples (p = 0.0655 r = 0.5727; Fig. 4E). Significantly, we found that baseline levels of Th17 cells were negatively correlated with the percentage of HIV-specific CD8^+^ T-cells that express the immunosuppressive PD-1 molecule (a marker of cell exhaustion) at later times (p = 0.0440 r = −0.6150; Fig. 4F). It is important to note that all these correlations were specific for the Th17 subpopulation as no relationship between CD8^+^ T-cell responses and Th1 levels were found (Supplementary Fig. S1).

The results described in this section suggest a direct relationship between Th17 baseline levels and protective HIV-specific CD8^+^ T-cell functionality (at both early as well as later times p.i), highlighting a potential impact of the maintenance of this CD4^ +^ T-cell subpopulation to preserve an effective anti-HIV immunity.

### Th17 levels were also correlated with other T-cell effector subsets

After the preceding results indicating a correlation between Th17 cells and HIV-specific CD8^+^ T-cells, we also considered interesting to evaluate the possible influence of Th17 levels on other potential T-cell effector functions. For this purpose, effector T-cells were phenotyped, after TCR polyclonal stimulation, as Th1 (CD4^+^ IFN-γ^+^), Tc1 (CD8^+^ IFN-γ^+^) and Tc17 (CD8^+^ IL-17^+^) following the protocol described in Materials and Methods.

**Figure 5:**
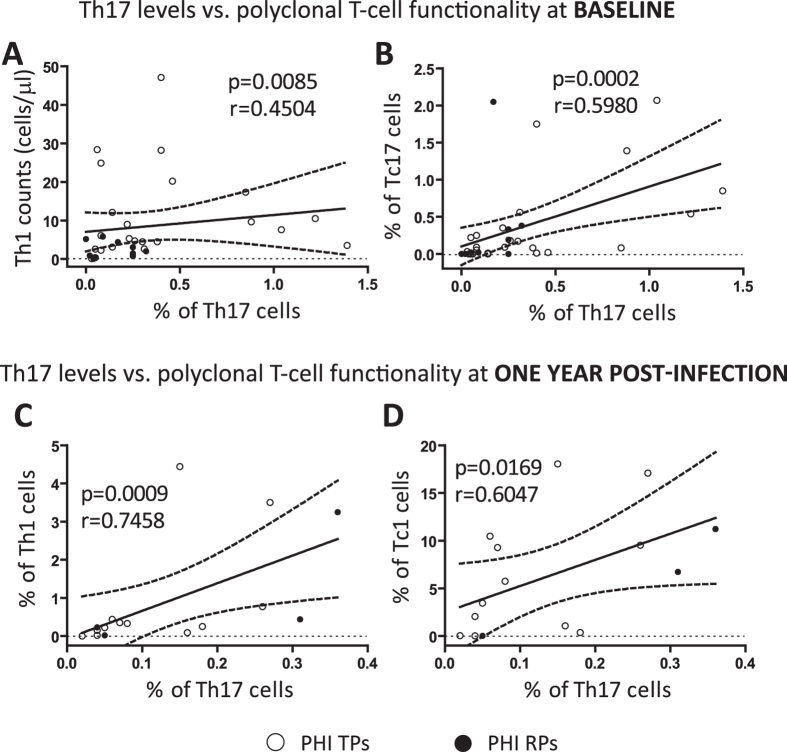
Correlations between the Th17 subset and other T-cell effector sub-populations measured upon polyclonal stimulation. Positive correlations between Th17 levels and polyclonal T-cell effector subsets found within PHI cohort: % of Th17 cells versus (vs.) Th1 (CD4^ + ^IFN-γ^ + ^) counts (**A**) and % of Tc17 (CD8^ + ^IL-17^ + ^) cells (**B**) both at baseline; % of Th17 cells vs. % of Th1 cells (**C**) and % of Tc1 (CD8^ + ^IFN-γ^ + ^) cells (**D**) both at one year p.i. Polyclonal T-cell effector sub-populations were determined by flow cytometry after 6 hours of stimulation with a mixture of anti-CD3/anti-CD28 antibodies as described in Materials and Methods. Symbols distinguish individual patients from the different sub-groups indicated in the figure. *PHI*: primary HIV infection cohort. *TPs*: typical progressors. *RPs*: rapid progressors. All *r* and *p* values correspond to Spearman’s correlations.

In Fig. 5 it can be seen that baseline Th17 frequencies in PHIs were directly correlated with Th1 counts (p = 0.0085 r = 0.4504; Fig. 5A) and also with proportions of Tc17 cells (p = 0.0002 r = 0.5980; Fig. 5B) both at baseline. When these analyses were performed in samples obtained at one year p.i, we also found a direct correlation between Th17 cells and frequencies of Th1 (p = 0.0009 r = 0.7458; Fig. 5C) and Tc1 (p = 0.0169 r = 0.6047; Fig. 5D) cells.

A further analysis revealed that the frequencies of Th1 and Tc17 cells tended to suffer a reduction during the first year p.i and, at this late time points, levels were significantly reduced compared to HDs. In contrast, Tc1 cells evaluated at one year p.i were not diminished neither in relation to HDs or baseline values (Supplementary Fig. S2). These results reinforce the concept that, during HIV infection, not only the Th17 but also other T-cell subsets with important effector functions are damaged.

In conclusion, the results described above indicated that the proportion of Th17 cells was correlated not only with HIV-specific CD8^+^ T-cell responses but also with other T-cell effector functions exerted by different T-cell populations.

### Th17 functionality and Th17/Treg ratio at later times post-infection also influence the quality of the specific concurrent anti-HIV CD8^+^ T-cell activities

**Figure 6:**
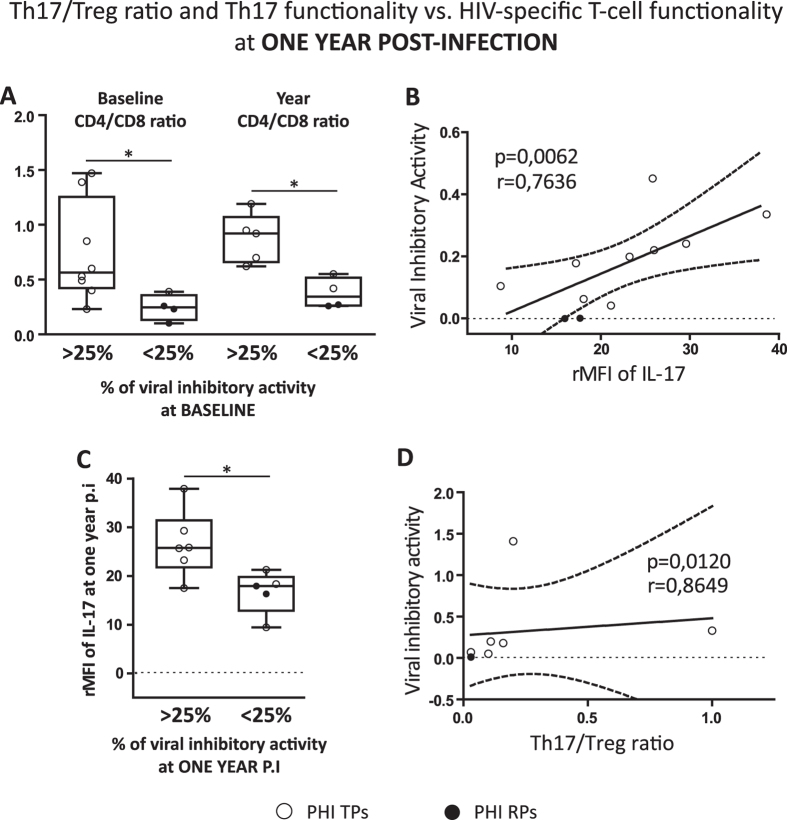
Influence of Th17 levels and Th17/Treg ratio on the quality of the HIV-specific CD8 T-cell responses at later times post-infection. Differences between PHI patients showing baseline viral inhibitory activity (VIA) higher or lower to 25 percent (> and <25%, respectively) regarding to their CD4/CD8 ratio at baseline and one year p.i (**A**). Correlation found between VIA versus (vs.) relative mean fluorescent intensity (rMFI) of IL-17 within PHI cohort at one year p.i (**B**). Comparison of PHI patients with VIA > and <25% at one year p.i regarding to their rMFI of IL-17 also at one year p.i (**C**). Correlation found between VIA versus (vs.) Th17/Treg ratio within PHI cohort at one year p.i (**D**). Symbols distinguish individual patients from the different sub-groups indicated in the figure. *PHI*: primary HIV infection cohort. *TPs*: typical progressors. *RPs*: rapid progressors. Boxes indicate median values with 25–75 percentiles and bars show the maximum and minimum values. The *p* values obtained are depicted as * p < 0.05 (**A** and **C**). All *r* and *p* values correspond to Spearman’s correlations (**B** and **D**).

Next, we proceeded with the analysis of the possible relationships between Th17 levels, Th17 functionality (rMFI of IL-17) and Th17/Treg ratio vs. relevant anti-HIV CD8^+^ T-cell activities at later times p.i within the PHI cohort. The importance of evaluating the *ex vivo* VIA of CD8^+^ T-cells is reflected by the fact that it was probed to be predictive of the rate of CD4^+^ T-cell decline during HIV infection^17^. Importantly, when PHIs were sub-divided considering the magnitude of their CD8^+^ T-cell VIA, we found that PHI subjects with higher baseline VIA (>25%) showed significantly higher CD4/CD8 ratio compared to PHIs with lower VIA (<25%) at both, baseline and one year p.i (Fig. 6A). These results highlight the prognostic value of this antiviral CD8^+^ T-cell function as it was previously reported for other patient cohorts^17^. Then, it was interesting to find that Th17 functionality (evaluating rMFI of IL-17 as indicator of IL-17 production) was positively correlated with VIA (p = 0.0062 r = 0.7636; Fig. 6B). And in this case when samples were subdivided by their VIA levels (**>**25% or <25%), we detected significant differences between both groups, finding that in those patients with a higher VIA, higher rMFI of IL-17 was also detected [25.8 (21.8–31.5) for VIA>25% vs. 17.9 (12.9–19.8) for VIA<25%; p = 0.0206; Fig. 6C]. Finally, we also found a direct correlation between Th17/Treg ratio and VIA (p = 0.0120 r = 0.8649; Fig. 6D). Thus, PHI patients with a higher preservation of the Th17/Treg ratio showed HIV-specific CD8^+^ T-cells with superior capacity to suppress HIV replication *in vitro*.

The results described in this section showed that at later times p.i both the functionality of Th17 cells and also the Th17/Treg ratio were related with the functional anti-HIV activity of the CD8^+^ T-cells (VIA).

## Discussion

In this study we analyzed the frequency of Th17 and Treg subsets, their ratio and correlation with clinical parameters and HIV-specific CD8^+^ T-cell responses during PHI.

Noticeably we found that, compared to HDs, the Th17/Treg ratio was significantly reduced in all HIV-infected patients, and within the PHI cohort we found a rapid reduction in the Th17/Treg ratio (in baseline samples). This finding is relevant when interpreted in the context of a previous study performed in the SIV model by Favre *et al.*^18^. That work showed that pathogenic SIV acute infection of pigtailed macaques was characterized by a rapid and marked selective depletion of Th17 cells and drop of the Th17/Treg ratio in blood and multiple tissues, indicating that an acute imbalance is related to SIV disease progression. The Th17/Treg ratio was significantly reduced even in ECs, and this reduction in the Th17/Treg ratio coincided with higher proportions of activated CD8^+^ T-cells in this group (compared to the normal levels found in HDs, Fig. 2D). In contrast to the many recent studies showing evidences of an activated innate and T-cell immune response in ECs^19^,^20^,^21^, we could only find one report that compared Th17/Treg ratio between ECs and HDs, in which a trend to lower Th17/Treg ratio in ECs can be appreciated^22^. In this context, our results suggest that the potential correlation between Th17/Treg imbalance and levels of immune activation that are sometimes present in ECs is a topic that merits further in-depth analyses.

Within the PHI cohort, we found a trend towards a Th17 reduction between baseline and one year p.i samples, however significant differences were not reached in line with previous studies^13^,^23^. However, at baseline we found a clear difference between RPs and TPs in relation to CD8^+^ T-cell activation, VL and Th17 counts. The pattern observed in RPs is in line with previous data of advanced HIV infection^8^,^24^ and more importantly, with the factors associated with severe symptomatic PHI previously described for the patients from the Grupo Argentino de Seroconversión study group^25^ and also for other HIV infected cohorts^26^. At early times p.i, preservation of the Th17 subset (evaluated by different means such as plasma IL-17 levels, percentage of Th17 cells and rMFI of IL-17) correlated with lower levels of activated CD4^+^ T-cells, and on the other hand with both higher CD4 counts and levels of MDC/CCL22. This β chemokine has been previously associated with HIV-protective activities^15^,^27^, although still some controversy exist regarding its role in inhibition of HIV replication depending on the sources of the infected cells, virus strains and chemokines employed in the *in vitro* studies performed^28^,^29^,^30^.

Afterwards, at later times p.i, higher Th17 levels remain positively correlated with clinical status (CD4 counts) and inversely correlated with baseline levels of the inflammatory sCD40L, a biomarker associated with disease during HIV/AIDS^16^ (Fig. 3E-F). Summarizing, findings related to the first part of our study indicated a clear relationship between a better clinical status with higher Th17 and lower Treg levels. As this last T-cell subset is known to suppress T-cell responses, negative correlations with levels of immune activation could be expected, however we found direct correlations with CD8^+^ T-cell activation and VL, and an inverse relationship with CD4 counts. These results are in line with recent studies which described direct correlations between Treg cells and T-cell activation^22^ or disease progression^23^, and inverse correlations with CD4 frequencies^22^, thus supporting the hypothesis of a detrimental role for this subset or the expansion of non-functional Tregs during HIV infection.

The main novel contribution of the present work relies on the analysis of the potential impact that the alterations recorded in the proportions and balance of Th17 and Treg subsets could have on the HIV-specific CD8^+^ responses with relevance on the antiviral HIV immunity during PHI. Our results revealed direct relationships between baseline Th17 levels and VIA against HIV and also with other key polyfunctional features (IFN-γ^+^/CD107_A/B_^+^) of the HIV-specific CD8^+^ T-cells at both early and later times p.i (Fig. 4), highlighting the potential prognostic value of the Th17 subset for the preservation of an effective HIV immunity. Importantly, we still detected positive correlations between Th17 functionality (rMFI of IL-17) and Th17/Treg ratio with VIA at one year p.i follow^-^up (Fig. 6).

In line with our findings, some previous reports also suggested the potential influence of the Th17 subset on the functionality of the HIV/SIV-specific T-cell responses. One of these works analyzed CD4^+^ T-cell restoration in GALT of HIV-infected patients, finding that an effective restoration was associated with an enhanced proportion of Th17 cells and polyfunctional HIV-specific T-cell responses^31^. Another study performed in the SIV model not only demonstrated that SIV replication is limited by the preexisting Th17 cell compartment and that a reduction in the Th17/Treg ratio was associated with higher VL, but also that these variables could shape and interact with T-cell mediated responses against the virus^32^. Consequently, animals with higher levels of Th17 cells had the highest SIV-specific CD4^+^ and CD8^+^ T-cell responses, and more importantly, specific CD4^+^ T-cell responses observed in animals with high Th17/Treg ratios were also more functional.

To further inquire into the possible relationship between Th17 levels and potential T-cell effector functions, we decided to also analyze other T-cell effector subsets after a polyclonal stimulation. Our results suggest that preservation of the Th17 cell compartment can be also important to preserve higher levels of other T-cell effector functions, potentially implicated with the T-cell capacity to confront other pathogen infections.

The Th17 and the Th17/Treg ratio preservation/deterioration observed in our study may have different consequences with relevant implications on the anti-HIV CD8^+^ T-cell functions. One of them is the loss of CD4^+^ T-cells that we found to be positively and negatively correlated with frequencies of Tregs and Th17 counts, respectively (Fig. 3). The progressive decline in the CD4 counts in HIV, as well as in other chronic viral infections, has been associated with the dysfunction of CD8^+^ T-cells justified by the reduction in the CD4^+^ T-cell help which is necessary for the CD8^+^ T-cell secondary expansion^33^,^34^. Also, the impact that high VL may have on T-cell dysfunction is an important factor to take into account^34^,^35^,^36^, as well as the presence of activated virus-specific CD8^+^ T-cells without an effector function (exhausted) as a mechanism of viral immune evasion^37^. In relation to these two last important factors, in this study we found that while Th17 levels were inversely correlated with VL, the percentage of Treg cells positively correlated with viral replication, in line with their direct relationship with higher frequencies of activated CD8^+^ T-cells. On the other hand, higher proportions of exhausted HIV-specific CD8^+^ T-cells (positive for PD-1) at later times p.i inversely correlated with baseline Th17 levels (Fig. 4F). Another plausible hypothesis which could explain the positive direct link between Th17 with CD8^+^ T-cell functionality, is the action exerted by interleukin 21 (IL-21), which is a pleiotropic cytokine expressed at high levels by Th17 cells and other activated CD4^+^ T-cells^38^. This cytokine exerts multiple functions, as to maintain long-term functional anti-viral CD8^+^ T-cells^33^,^39^,^40^,^41^ and an adequate pool of fully functional Th17 cells^42^,^43^. Thus, in a recently published study administration of IL-21 to SIV-infected macaques was accompanied by higher expression of perforin and granzyme B in total and SIV-specific CD8^+^ T-cells (better functionality) and higher levels of intestinal Th17 cells^44^. These previous observations raise the possibility that our findings, showing a positive correlation between HIV-specific CD8^+^ T-cell responses and Th17 levels and functionality (rMFI of IL-17), could be explained by inferring that higher levels of Th17 cells would translate into higher IL-21 production.

It must be notice that despite the novelty of our findings, one of the constraints of the present study was the limitation in the samples available to perform the multiple evaluations. Thus, although 40 PHI subjects were enrolled, not all the samples rendered sufficient cell numbers to perform the complete set of assays. On the other hand, another limitation is that all the analyses were performed in the peripheral blood compartment, and it must be taken into account that the Th17 T-cell subset exerts its main functions at mucosal level.

A relevant topic considered is the effect of ART on the reestablishment of Th17 proportions and Th17/Treg balance, as different previous studies have reported that after ART treatment the restoration of Th17 numbers and functionality^31^,^45^ as well as the Th17/Treg balance may not be fully recovered^46^. In this context a recent important study highlighted the relevance of the initiation of ART during early acute HIV infection, demonstrating its benefits to preserve the mucosal Th17 function and to reverse the HIV-related immune activation^47^. Thus, considering these previous data and the results from the present study it will be important to evaluate in the future the potential impact of the ART treatment on Th17 and Th17/Treg balance restoration and its influence on the re-establishment of the quality of the CD8+ T-cell responses after the treatment.

In summary, the findings described in this study suggest for the first time that an interaction between Th17 and Th17/Treg balance with the functionality of the HIV-specific CD8^+^ T-cell response may exist. Overall, to our knowledge this is the first report from a South-America cohort of HIV subjects to address the interplay of Th17/Treg cells with disease progression and specific CD8^+^ T-cell functionality.

## Materials and Methods

### Study population

A total of 84 individuals participated in this study: 14 healthy HIV-seronegative donors [HDs; voluntary blood donors from the Sanatorio Dr. Julio Mendez blood bank (Buenos Aires, Argentina), all of them individuals older than 18 years that completed and passed a survey on blood donation and were screened for serological markers before being accepted as donors] and 70 HIV-infected patients, of whom 40 were enrolled during PHI, 17 were chronically infected (Chronics) and 13 were defined as ECs (Table 1). PHI subjects were enrolled by the Grupo Argentino de Seroconversión Study Group under the following inclusion criteria^25^: (i) detection of HIV-1 RNA or p24 antigen with a simultaneous negative or indeterminate Western blot (WB) assay or (ii) positive WB assay with a negative test within the previous 6 months. For some analyses, the PHI group was further sub-divided into 2 subgroups whether their CD4 counts dropped below 350 cells/μl during the first year p.i or not, denoted as rapid (RPs) and typical (TPs) progressors, respectively. Chronics were defined as individuals with established HIV-1 infection for more than 3 years, high VL (>10.000 HIV-1 RNA copies/ml plasma), and that were ART naïve at the time of enrollment. ECs were defined as persons HIV-infected for more than 5 years with undetectable VL (<50 HIV-1 RNA copies/ml plasma) without ART, CD4 counts >450 cells/μl and with no records of opportunistic infections and/or AIDS-related diseases. The study was reviewed and approved by two institutional review boards: *Comité de Bioética y Docencia e Investigación Hospital General de Agudos Juan A. Fernández (Buenos Aires, Argentina; protocolo CODEI 201115, 29/08/2011)* and *Comité de Bioética Fundación Huésped (Buenos Aires, Argentina).* All HIV-infected participants provided written informed consent accepting to participate in this study and the methods applied were carried out in accordance with the approved guidelines.

### Human samples

Blood samples were collected at enrollment on tubes with EDTA and centrifuged to separate plasma, which was stored at −80 °C until use. Peripheral blood mononuclear cells (PBMCs) were isolated by Ficoll-Hypaque density gradient centrifugation (Amersham, Sweden) and cryopreserved. For those PHI patients that remained ART naïve, additional samples were obtained at a median of 330 days p.i (see Table 1). For Chronics, ECs and PHIs, plasma VL (branched-DNA, Versant HIV-1 RNA 3.0 assay; Siemens Healthcare) and CD4/CD8 counts (flow cytometry double platform, BD FACSCanto; BD Biosciences) were determined.

Subsequent functional assays were performed, according to sample availability, using only thawed cells that showed >95% viability after overnight rest in complete RPMI medium [RPMI-1640 (Sigma-Aldrich, USA) supplemented with 10% fetal bovine serum (Gibco, USA), 2 mM L-glutamine (Sigma-Aldrich, USA), 100 U/ml penicillin (Sigma-Aldrich, USA), 100 mg/ml streptomycin (Sigma-Aldrich, USA), and 10 mM HEPES (Gibco, USA)].

### Phenotyping of Th17, Treg and other T-cell subsets of interest

Different subsets were evaluated by flow cytometry using thawed and overnight rested PBMCs dispensed in U-bottom 96-well plates (between 5 × 10^5^ and 10^6^ cells/well were used, depending on sample availability). All fluorochrome-conjugated, isotype and co-stimulatory antibodies (Abs) used in this study were obtained from BD Biosciences (USA).

Treg cells (defined as CD4^+^ CD25^+^ FoxP3^+^) were evaluated in unstimulated PBMCs, stained for 30 minutes (min) at 4 °C with LIVE/DEAD Fixable NEAR-IR (Invitrogen), anti-CD3-PerCP, anti-CD4-FITC and anti-CD25-APC. For intranuclear staining, the Human FoxP3 Staining Kit (BD Biosciences) was used according to manufacturer’s instructions. Briefly, after the fixation and permeabilization steps, cells were incubated for 30 min at 4 °C with anti-FoxP3-PE. Isotype-matched APC- and PE-conjugated non-specific Abs were used in each sample in order to accurately set FoxP3 and CD25 negative populations (see Supplementary Fig. S3).

Th17 cells (defined as CD4^+^ IL-17^+^) were identified by intracellular cytokine staining (ICS) after 6 hours (hs) of polyclonal stimulation at 37 °C with anti-CD3 and anti-CD28 Abs (10 ng/ml each) and monensin (0.7 μl/ml; GolgiStop, BD Biosciences). This assay also allowed the simultaneous detection of the following T-cell subsets: Th1 (CD4^+^ IFN-γ^+^), Tc17 (CD8^+^ IL-17^+^) and Tc1 (CD8^+^ IFN-γ^+^). Briefly, surface staining consisted of 30 min incubation at 4 °C with LIVE/DEAD Fixable NEAR-IR (Invitrogen), anti-CD3-PECy7, anti-CD4-PerCP and anti-CD8-APC. Then, ICS was performed following the instructions of the Cytofix/Cytoperm kit (BD Biosciences), incubating 30 min at 4 °C with anti-IL-17A-PE and anti-IFN-γ–FITC. Unstimulated controls (medium only) were included and frequencies presented correspond to background-subtracted results for each patient (see Supplementary Fig. S3). Samples with a background higher than 0.05% were retested using a new vial of frozen cells, when available.

Additionally, Th17 functionality was evaluated as the density of expression of IL-17 in Th17 cells (MFI values on a logarithmic scale) relative to the MFI of IL-17 in total CD4^+^T-cells in each sample as follows: PE MFI_Th17_/PE MFI_total TCD4+_.

All samples were immediately acquired in a 2-laser, 6-color BD FACSCanto flow cytometer and analyses were performed using the BD FACSDiva software. Instrument settings and fluorescence compensation were performed for each day of testing using unstained and single-stained samples. The same initial gating strategy was applied in all flow cytometry assays (see Supplementary Fig. S3). First, small lymphocytes (between 250.000 and 80.000 events) were selected in a plot of forward scatter (FSC) vs. side scatter (SSC). Then, FCS area (FSC-A) vs. height (FSC-H) was constructed to remove doublets. Dead cells were then excluded by the LIVE/DEAD fluorescence. Subsequently, CD3^+^ CD4^+^ and CD3^+^ CD8^+^ events were gated in a CD3 vs. CD4 or CD8 dot plot. Finally, for selection of Th17 (as well as Th1, Tc17 and Tc1) the corresponding CD4 (or CD8) vs. IL-17 (or IFN-γ) were constructed. For Treg evaluation, CD4 vs. CD25 and CD4 vs. FoxP3 dot plots were constructed and the “derived gate tool” was used to accurately and automatically determine the double positive CD25^+^ FoxP3^+^ population.

### Immune activation

Immune activation was defined as the percent of CD38^+^ HLA DR^+^ T-cells (CD4 or CD8) and analyzed by flow cytometry. For this, thawed and overnight rested PBMCs were stained for 30 min at 4 °C with LIVE/DEAD Fixable NEAR-IR (Invitrogen), anti-HLA-DR-FITC, anti-CD4-PerCP, anti-CD38-APC, anti-CD3-PeCy7 and anti-CD8-PE (all Abs obtained from BD Biosciences). Data acquisition and analysis was performed using the BD FACSDiva software. Initial gating was performed as described above. Isotype-matched FITC- and APC-conjugated non-specific Abs were used in each sample to set HLA-DR and CD38 negative populations. The “derived gate tool” was used to accurately and automatically determine the double positive CD38^+^ HLA-DR^+^ population.

### Virus inhibitory activity (VIA) and CD8^+^ T-cell bi-functionality

The *ex vivo* ability of CD8^+^ T-cells to inhibit HIV-1 replication in primary autologous CD4^+^ T-cells (VIA) and the capacity of HIV-specific CD8^+^ T-cells to simultaneously produce IFN-γ and degranulate (evidenced by CD107_A/B_ mobilization) upon HIV-peptides stimulation were evaluated following the same protocols previously published^11^. Examples of the gating strategy applied to detect bi-functional cells by flow cytometry are illustrated in Supplementary Fig. S4.

### Exhaustion of the HIV-specific CD8^+^ T-cell response

The expression of PD-1 marker was evaluated by flow cytometry on HIV-specific CD8^+^ T-cells as recently published^48^.

### Quantification of plasma soluble factors

Simultaneous determination of the following 39 cytokines and chemokines was performed in plasma samples from a subset of 20 PHI subjects (at baseline only) using Luminex technology (MILLIPLEX MAP Human Cytokine/ Chemokine; Millipore): EGF, eotaxin, FGF-2, Flt-3 ligand, fractalkine, G-CSF, GM-CSF, GRO, IFN-α2, IFN-γ, IL-1α, IL-1β, IL-1rα, IL-2, IL-3, IL-4, IL-5, IL-6, IL-7, IL-8, IL-9, IL-10, IL-12 (p40), IL-12 (p70), IL-13, IL-15, IL-17, IP-10, MCP-1, MCP-3, MDC (CCL22), MIP-1α, MIP-1β, sCD40L, sIL-2Rα, TGF-α, TNF-α, TNF-β, VEGF. Samples were processed and analyzed as described by Giavedoni^49^.

### Data analysis

For PHI patients, the estimated time p.i was calculated as described (see^25^ and Table 1). Statistical analyses were performed using GraphPad Prism 5 (Graph-Pad Software). All data except Log_10_ VL were analyzed using nonparametric statistics. Kruskal-Wallis and two-tailed Wilcoxon and Mann-Whitney tests were used to compare intra- and intergroup variables, respectively. Correlations were determined using Spearman’s rank test. All tests were considered significant if the *p* value obtained was less than 0.05.

## Additional Information

**How to cite this article**: Falivene, J. *et al.* Th17 and Th17/Treg ratio at early HIV infection associate with protective HIV-specific CD8^+^ T-cell responses and disease progression. *Sci. Rep.* **5**, 11511; doi: 10.1038/srep11511 (2015).

## Supplementary Material

Supplementary Information

## Figures and Tables

**Table 1:** Characteristics of HIV-infected individuals enrolled for the study.

Group	Sample time points, n° of subjects	Estimated dpi^a^, median (IQR)	Age (years), median (IQR)	Log_10_VL^b^, mean± SD	CD4 counts^c^, median n° of cells/μl (IQR)	CD8 counts^c^, median n° of cells/μl (IQR)	CD4/CD8 ratio, median (IQR)
**PHI cohort**	Baseline (n = 40)	75 (50–120)	32 (23–37)	4.52 ± 1.10	577 (388–681)	1099 (767–1593)	0.50 (0.27–0.79)
	Year (n = 22)	330 (300–360)	–	4.27 ± 0.84	480 (396–660)	845 (594–1200)	0.57 (0.35–0.91)
**PHI RPs**	Baseline (n = 11)	55 (30–98)	37 (27–40)	4.98 ± 0.85	282 (222–379)	1324 (930–1771)	0.23 (0.12–0.33)
	Year (n = 5)	300 (230–370)	–	4.01 ± 1.14	316 (178–358)	961 (540–1219)	0.27 (0.25–0.41)
**PHI TPs**	Baseline (n = 29)	90 (60–120)	30 (23–36)	4.35 ± 1.14	603 (515–741)	1021 (634–1420)	0.60 (0.40–0.87)
	Year (n = 17)	330 (300–360)	–	4.34 ± 0.63	575 (443–680)	769 (592–1217)	0.70 (0.44–0.94)
**ECs**	≥5 years (n = 13)	–	37 (33–44)	<1.70	599 (559–894)	618 (287–802)	1.54 (0.61–2.38)
**Chronics**	≥3 years (n = 17)	–	34 (25–36)	4.71 ± 0.70	141 (11–395)	1009 (540–1265)	0.19 (0.03–0.37)

*PHI:* primary HIV infection. *RPs:* rapid progressors. *TPs:* typical progressors. *ECs:* elite controllers. *dpi:* days post-infection. *VL:* viral load. *IQR:* inter quartiles. *SD:* standard deviation. RPs and TPs distinguish PHIs that had CD4 counts below or above 350 cells/μl during the first year post-infection, respectively.

^a^In symptomatic patients, estimated as 14 days before the onset of symptoms. In asymptomatic patients, estimated as the midpoint between the last negative and the first positive test or one month before the date of the indeterminate or negative Western blot assay^25^.

^b^Versant HIV-1 RNA 3.0 assay, Siemens. Lower and upper detection limits are 50 and 500.000 RNA copies/ml, respectively (1.7 and 5.7 log_10_).

^c^Flow cytometry double plataform, FACSCanto, BD Biosciences.
