# Supplementary material for: Th17 and Th17/Treg ratio at early HIV infection associate with protective HIV-specific CD8+ T-cell responses and disease progression
Source: Sci Rep. 2015 Jun 23;5:11511. doi: 10.1038/srep11511 (PMC4477236; doi:10.1038/srep11511)
Supplement: Supplementary Information [file srep11511-s1.pdf]

Th17 and Th17/Treg ratio at early HIV infection associate with protective HIV-specific CD8$^+$ T-cell responses and disease progression

Juliana Falivene[1], Yanina Ghiglione[1], Natalia Laufer[1,3], María Eugenia Socías[2], María Pía Holgado[1], María Julia Ruiz[1], Cynthia Maeto[1], María Inés Figueroa[2], Luis D. Giavedoni[4], Pedro Cahn[2,3], Horacio Salomón[1], Omar Sued[2], Gabriela Turk[1], María Magdalena Gherardi [1*].

[1]Instituto de Investigaciones Biomédicas en Retrovirus y SIDA (INBIRS), Universidad de Buenos Aires-CONICET, Buenos Aires, Argentina. [2]Fundación Huésped, Buenos Aires, Argentina. [3]Hospital J.A. Fernández, Buenos Aires, Argentina. [4]Department of Virology and Immunology, Southwest National Primate Research Center, Texas Biomedical Research Institute, San Antonio, TX, USA.

RUNNING TITLE: Interplay of Th17 and Treg with HIV-CD8$^+$ T-cells

[*]**CORRESPONDING AUTHOR**

Dr. M. Magdalena Gherardi
Instituto de Investigaciones Biomédicas en Retrovirus y SIDA INBIRS (ex Centro Nacional de Referencia para el SIDA), Universidad de Buenos Aires- CONICET
Paraguay 2155 Piso 11
C1121ABG - Buenos Aires, Argentina
TE +54 11 4508 3689 ext 117
FAX +54 11 4508 3705
e-mail: mgherardi@fmed.uba.ar

SUPPLEMENTARY FIGURE S1

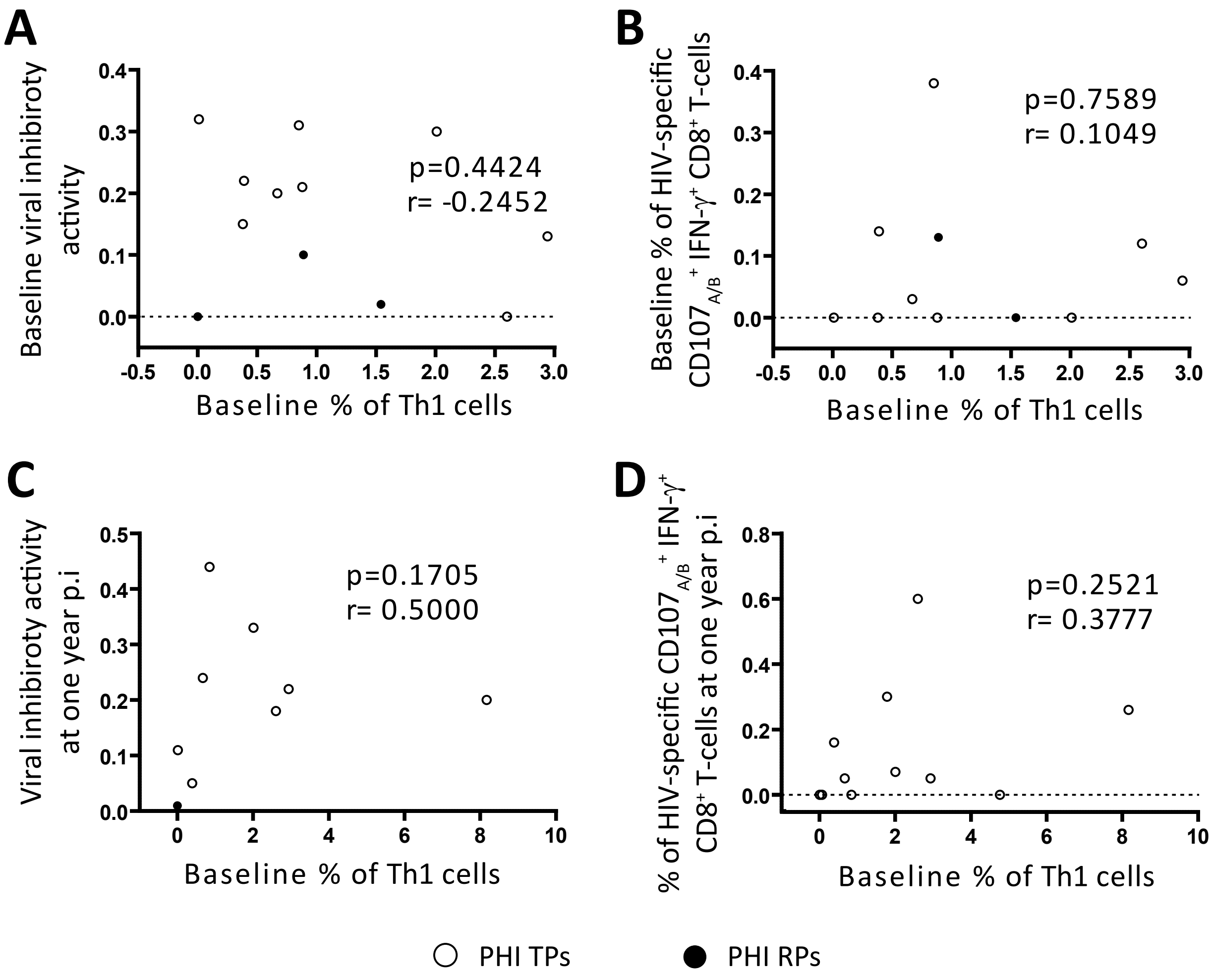

Supplementary Figure S1: Th1 subset does not correlate with HIV-specific CD8 T-cell responses. Th1 cells (defined as CD4+ IFN-γ+) were determined by flow cytometry after polyclonal stimulation of PBMCs as described in Materials and Methods. The figure shows that no correlations exist between Th1 frequencies at baseline and HIV-specific CD8 T-cell responses at both, baseline (**A** and **B**) and one year p.i (**C** and **D**). Particularly, baseline % of Th1 versus (vs.) viral inhibitory activity (VIA, **A** and **C**) and % of HIV-specific $CD107_{A/B}$+ IFN-γ+ CD8 T-cells (**B** and **D**) are shown at both time points. The same is observed when baseline Th1 counts and Th1 levels at one year p.i are analyzed (data not shown). HIV-specific CD8 functionality was determined with two different assays: one of them allowed the evaluation of CD8 T-cells with the capacity to degranulate and simultaneously secrete IFN-γ upon HIV-peptides stimulation by flow cytometry, the other measured the overall CD8 T-cell capacity to inhibit *in vitro* HIV-1 replication in autologous CD4 T-cells. These assays are described in detail in our previous publications (see Turk et al. and Ghiglione et al.). Symbols distinguish individual patients. PHI: primary HIV infection cohort. TPs: typical progressors. RPs: rapid progressors. All *r* and *p* values correspond to Spearman's correlations.

SUPPLEMENTARY FIGURE S2

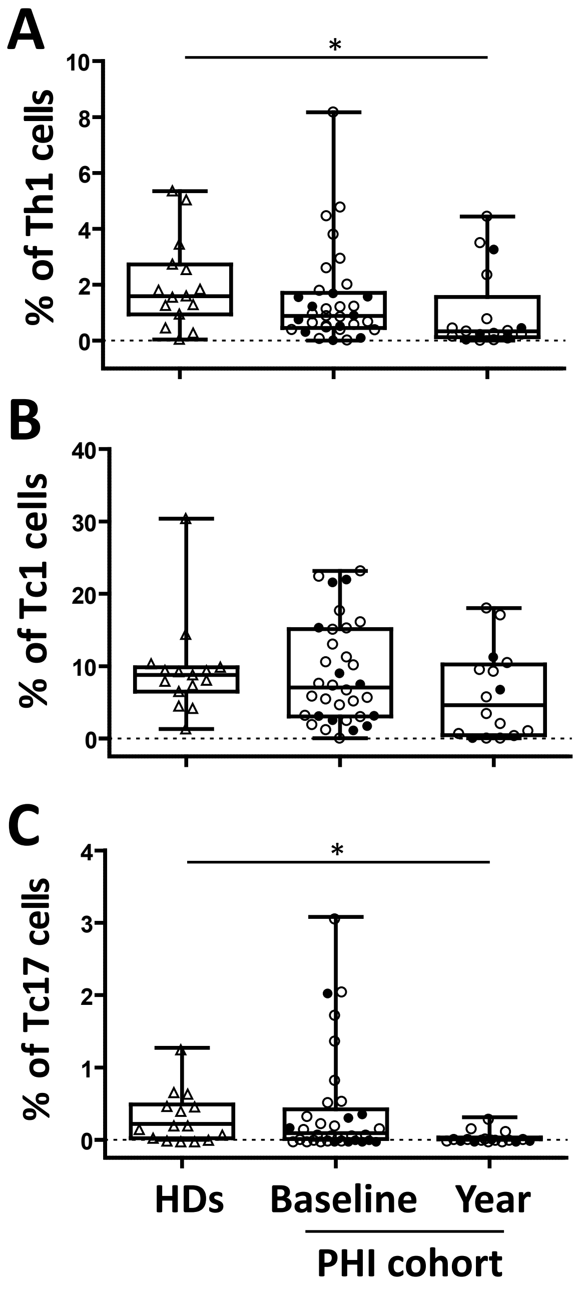

Supplementary Figure S2: Evaluation of the Th1, Tc1 and Tc17 subsets during primary HIV infection. PBMCs were stimulated for 6 hours with anti-CD3/anti-CD28 or medium alone (background control) prior to intracellular staining. Background subtracted values are shown for: Th1 (CD4+ IFN-γ+) (**A**) and Tc1 (CD8+ IFN-γ+) (**B**) populations, and Tc17 (CD8+ IL-17+) cells (**C**). Boxes indicate median values with 25-75 percentiles and bars show the maximum and minimum values. Symbols represent individual patients within each group: Healthy donors (HDs) and primary HIV infection (PHI) cohort at baseline and one year p.i follow up (white circles depict typical progressors, or TPs with CD4 T-cell counts above 350 cells/μl during the first year p.i, and black circles rapid progressors, or RPs with CD4 T-cell counts below 350 cells/μl during the first year p.i). The *p* values obtained are depicted as * $p<0.05$.

SUPPLEMENTARY FIGURE S3

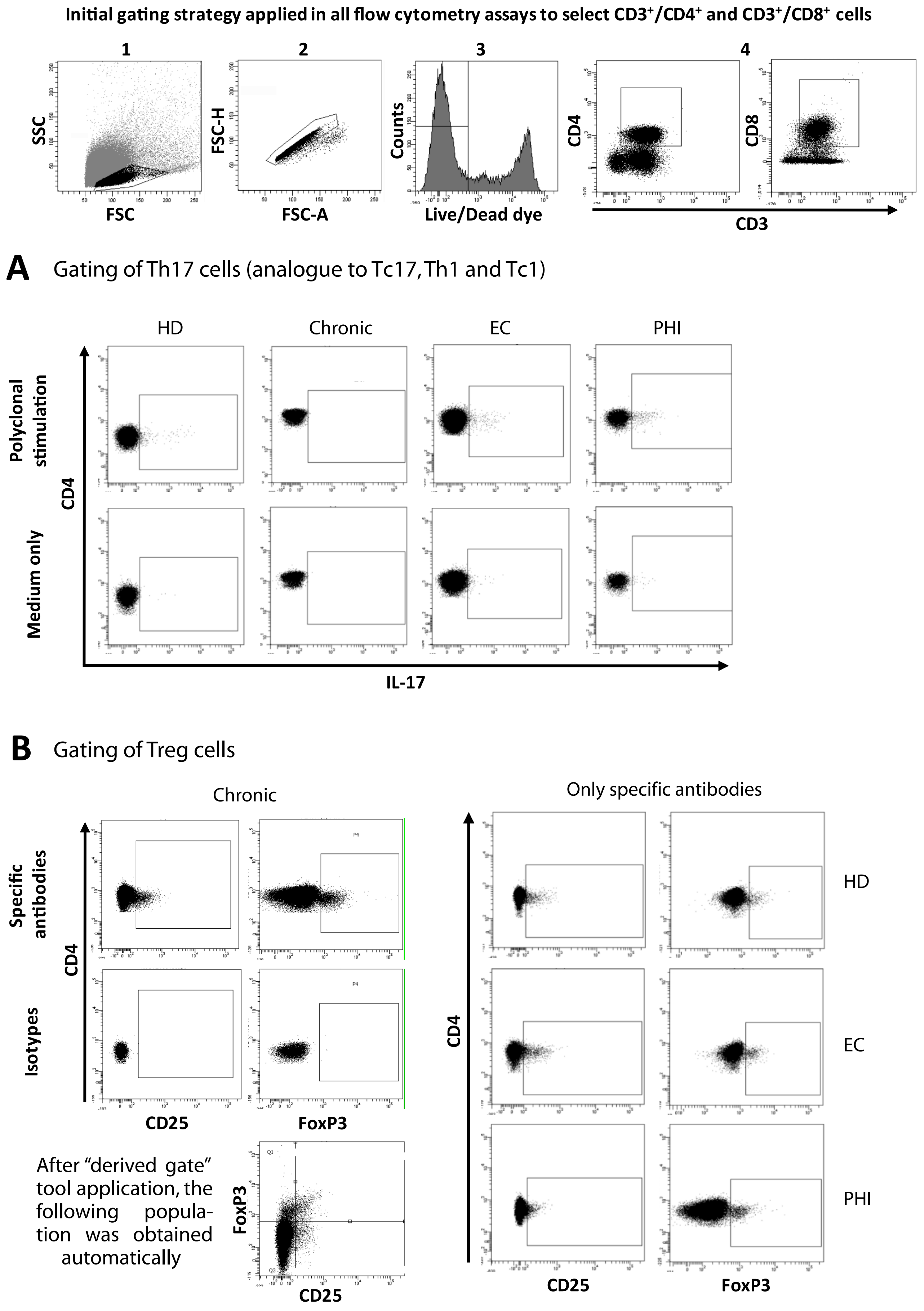

Supplementary Figure S3: Schemes and examples of the gating strategies applied for evaluation of Th17 and Treg subsets using a FACSCanto II flow cytometer. As depicted in the upper line of the figure, in all the assays a first gating was performed on small lymphocytes in a plot of forward scatter (FSC) versus (vs.) side scatter (SSC) (**1**). Then, FCS area (FSC-A) vs. height (FSC-H) dot plot was constructed to remove doublets (**2**). An histogram allowed to exclude dead cells with the LIVE/DEAD fluorescence (**3**). Finally, CD3+ CD4+ (**4**, left) or CD3+ CD8+ (**4**, right) events were gated in the corresponding dot plots. Scheme **A** shows the strategy used to identify Th17 cells. After polyclonal stimulation (upper panels), the frequencies of IL-17+ cells were determined after background subtraction (medium only, lower panels). For determination of Th1, Tc1 and Tc17 subsets the same strategy was applied, detecting IFN-γ or IL-17 within CD4 or CD8 T-cells. Scheme **B** shows the gates applied to obtain Treg cells. In this case, the cells were directly assayed without any exogenous *in vitro* stimulation. For this reason, the setting of the negative populations was determined with matched isotype controls, which consisted of cells stained with the conjugated antibodies to surface molecules (CD3 and CD4) and isotype controls corresponding to surface (CD25) and intranuclear (FoxP3) markers of interest. The “derived gate tool” available in the FACSDiva software was used to accurately and automatically determine the double positive CD25+ FoxP3+ population, in contrast to manual/visual double positive dot plot strategy, as illustrated. Both schemes show representative dot plots obtained from one patient of each group for illustrative purposes. HD: healthy donor. EC: elite controller. PHI: primary HIV infection patient (baseline sample).

SUPPLEMENTARY FIGURE S4

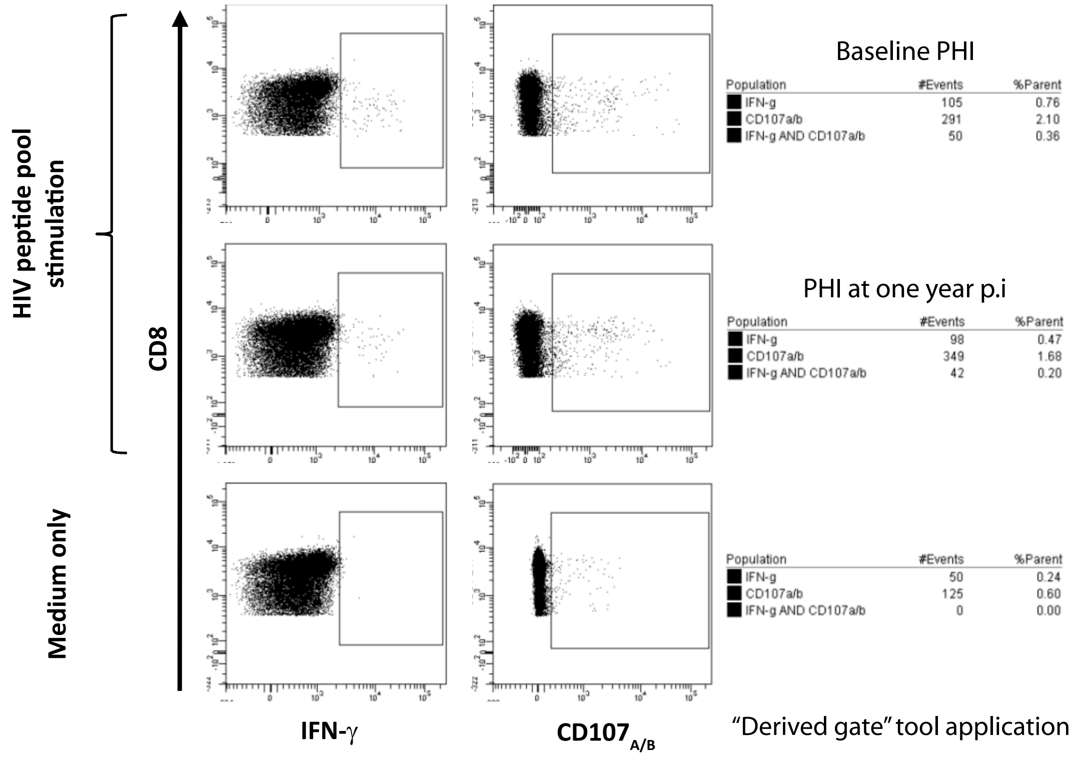

Supplementary Figure S4: Examples of the gating strategy applied for evaluation of HIV-specific polyfunctional IFN-γ^+ CD107$_{A/B}$$^+$ CD8$^+$ T-cells using a FACSCanto II flow cytometer. PBMCs were stimulated in the presence of a pool of HIV peptides or medium alone (background control) for 6 hours at 37ºC as described in our previous work (Turk et.al.). The same initial gating strategy described in Fig. S3 allowed to select live CD3$^+$ CD8$^+$ cells in these experiments. Representative dot plots obtained from one PHI (primary HIV infection) patient at baseline and one year p.i follow-up, indicating the gates applied to obtain HIV-specific polyfunctional IFN-γ^+ CD107$_{A/B}$$^+$ CD8$^+$ T-cells are shown. The "derived gate" tool available in the FACSDiva software was used to accurately and automatically determine the double positive population.
